# Preparedness for mass gatherings: rescue and emergency medical services’ workloads during mass gathering events

**DOI:** 10.1186/s13049-022-01003-7

**Published:** 2022-03-05

**Authors:** Anssi Koski, Jukka Pappinen, Anne Kouvonen, Hilla Nordquist

**Affiliations:** 1 South-Eastern Finland University of Applied Sciences, 48220 Kotka, Finland; 2 Faculty of Medicine, University of Helsinki, 00014 Helsinki, Finland; 3 Faculty of Health Sciences, University of Eastern Finland, PO Box 1627, 70211 Kuopio, Finland; 4 Faculty of Social Sciences, University of Helsinki, 00014 Helsinki, Finland; 5 Centre for Public Health, Queen’s University Belfast, Belfast, BT12 6BA UK

**Keywords:** Preparedness, Mass gatherings, Emergency medical services, Rescue service, Event organizers

## Abstract

**Background:**

Mass gathering (MG) events may cause delayed emergency responses via various mechanisms and strain the resources of local emergency services. Therefore, preparedness, including adequate pre-planning and sufficient resourcing during MG events, is vital. The aim of this retrospective register study was to investigate the impact of MG events on the workload of rescue and emergency medical service (EMS) personnel during events to enable more precise and sufficient deployment of these authorities’ operative resources.

**Methods:**

The data from Finland covered of 25,124 EMS and rescue service missions during a three-year period (2015–2017), including data from nine MG events and reference material for the same weekdays two weeks before and after the event. The data were analysed through statistical and geospatial analyses.

**Results:**

Our findings showed that missions increased in most events included in this study. Analysis of the missions’ reasons showed that the categories of violence, traffic accidents and other accidents and injuries increased during events, with violence-related missions showing the highest relative risk (RR 1.87, 95% CI 1.43–2.44). In the four-grade (A–D) urgency grading, the analysis showed an increase in category C missions and a decrease in non-urgent category D missions. The analysis indicated an increase in missions during the evening and night-time. The geospatial analysis revealed dense hotspots of missions in the vicinity of the event area.

**Conclusion:**

The workload for EMS and rescue service personnel increases during MG events. Most of the increase is allocated to EMS staff, peaking in evening and night hours. The geospatial analysis showed hotspots of missions on the outskirts of the actual event area during events; thus, the workload can also increase for those authority resources that are not directly allocated to the event. Detailed information regarding workloads is valuable for the authorities that are responsible for resource planning and preparedness for MG events. Replicating the study internationally would improve the methodology for the future.

**Supplementary Information:**

The online version contains supplementary material available at 10.1186/s13049-022-01003-7.

## Introduction

Mass gathering (MG) events may increase the workloads of local emergency services and healthcare facilities [1–4]. The World Health Organization criteria for MGs is that they are organized or spontaneous events where attendance is sufficient to strain local planning and response resources [5]. According to earlier studies, an increased workload during MG events especially affects emergency medical services (EMS) [1, 6–8]. The workload for rescue services seems to be concentrated mainly in the pre-event phase, focusing on the emergency planning process [6]. Previous research has shown that MG environmental characteristics that influence workload include on-site medical care [9–19], the event type, location and duration [20, 21] and the weather conditions [22, 23]. In addition, participant-related factors, such as drug and alcohol use and crowd demographics and behaviour, can have an impact on medical workload [3, 13, 15, 21, 24, 25].

An ability to predict workload provides valuable information for EMS and rescue service resource planning during MG events. In prior studies, different approaches, including retrospective [2, 3, 17, 20, 22–24, 26–31] and predictive [14, 21, 32–35] investigations of events, have been used. Overall, the previous literature on utilizing data on MGs is strongly dominated by healthcare. In this study, we included both EMS and rescue services and utilized geospatial analysis, thus bringing a new approach to this research area. Accurate information about workload during an event enables more precise resource and preparedness planning.

MG events in Finland occur mainly during summer months and across the country [36]. The population and major cities of Finland are highly concentrated in the southern and western parts of the country [37]. A large MG event in a relatively small town may cause a significant burden on the local EMS and rescue services.

The aim of this study was to investigate EMS and rescue service workloads during MG events. The specific objectives were to examine (1) if EMS and rescue service workload increases during MGs; (2) how time of day affects workload during the event; and (3) what the geospatial distribution of the workload during the event is.

## Methods

### Study setting

In this study, the workload of EMS and rescue services was investigated for nine MG events by comparing the mission data during these events to the reference data of the same weekdays two weeks before and after the event.

### Data collection

The material used in the present study consists of Emergency Response Centre mission (EMS and rescue service) data from a three-year period (2015–2017) during nine MG events in Finland. The data contain event data and reference mission data from the same weekdays as the event as well as two weeks before and two weeks after the event. The time period included 97 event days and 388 reference days. The MG events selected for this study were events held in various sized settlements, including the biggest cities—Helsinki, Tampere, Oulu and Turku—and smaller rural towns, i.e. Pori, Joensuu, Seinäjoki, Kotka and Jämsä. The events took place in different parts of the country, apart from the north. The duration of the events ranged from two to nine days.

### Eligibility criteria

The selection criteria for the MGs were summer as the time of the year, an outdoors venue and a live music event.

### Data sources, measurements and analysis

The data contain city/town, type of mission, urgency, date, time and coordinate information for each EMS or rescue service mission. EMS transfer missions between healthcare facilities were excluded. The data included 25,124 individual missions, of which 23,957 were EMS missions and 1167 were rescue service missions. EMS units respond to, for example, in traffic accidents and house fires, despite the responsible authority in command being the rescue service. Thus, the data were analysed in five mission categories that included both authorities: other accident/injury, traffic accident, violence, cardiac arrest and other diseases.

Missions are categorized according to their urgency. The urgency category affects the response to a specific mission. In the initial response, most high-risk missions include additional resources, such as first responders or helicopter-based emergency medical service (HEMS) units in addition to conventional EMS units. The urgency category also determines the initial response and the type and number of units of the rescue service. The urgency categories (ABCD) in EMS missions are defined in Finnish legislation under § 6 of the Social and Health Ministry decree on Emergency Medical Services 585/2017 (Table 1).

**Table 1:** Dispatch criteria

Mission category	Response	Level of risk		Situational assessment	
		EMS	Rescue	EMS	Rescue
A	Lights and sirens	High risk	High risk	Immediate threat for vital life functions	Immediate saving of a person or environment of great value of assets
B	Lights and sirens	Propable high risk	Propable high risk	No certanity of immediate threat for vital life functions	Unconfirmed possible lifesaving or major additional damage preventing
C	Urgent	Require swift assessment	Static accident	Stable vital life functions	Assessed not to result immediate additional damage
D	Non-urgent	Require assessment	No immediate action	Stable vital life functions	Response in approppriate or agreed date

The statistical analysis was conducted using Excel software, and the geospatial analysis was carried out with ArcGIS Pro for Windows, version 2.6.2 (Esri Inc.). Geospatial analysis combines coordinate data and geographical information in a visual display format. The mission information was processed on a map background with ArcGIS and displayed as heat maps, which indicate call density hotspots in the area. In the statistical analysis, relative risk (RR) with 95% confidence intervals (CIs) was calculated for the event locations, mission causes and urgency categories. P-values were calculated from the RRs to determine the statistical significance of the findings.

## Results

Categorized and classified by the responsible authority, the vast majority of the combined missions during the events (n = 5949) were EMS missions (n = 5617), and the rest were rescue service missions (n = 332). The operative workload of the EMS and the rescue services during MG events increased in 8 out of 9 events included in this study, and the RR was above 1 for all events, except those that took place in the capital city, Helsinki. The workload increase was statistically significant in 6 out of 9 events (Table 1). The event-specific investigation showed that the RR for an event was lowest (RR 0.97, 95% CI 0.77–1.22) in Helsinki (with the largest population) and highest in the town of Jämsä (with the smallest population) in Central Finland (RR 1.75, 95% CI 1.30–2.37) (Table 2). The total missions per day in the reference data of the same geographical (spatial) area were compared with the mission count during the event.

**Table 2:** Event-specific workload analysis

Event location (inhabitants)	Event n	Reference n	n/day event	n/day reference	n ± change/day during event	95% CI upper	95% CI lower	RR	Sig.
Helsinki (656 250)	1746	7180	194.0	199.44	− 5.44	1.22	0.77	0.97	0.823
Oulu (207 717)	600	1821	100	75.88	+ 24.12	1.68	1.04	1.32	< 0.05
Tampere (241 672)	527	1896	87.83	79.00	+ 8.83	1.42	0.87	1.11	0.399
Turku (194 244)	781	2365	86.78	65.69	+ 21,09	1.67	1.04	1.32	< 0.05
Joensuu (76 833)	415	1090	46.11	30.28	+ 15.83	1.95	1.19	1.52	< 0.01
Pori (83 676)	1063	2584	39.37	23.90	+ 15.47	2.08	1.30	1.65	< 0.01
Kotka (51 603)	422	1116	32.46	21.46	+ 11	1.94	1.18	1.51	< 0.01
Seinäjoki (64 335)	259	813	28.78	22.58	+ 6.2	1.66	0.98	1.27	0.070
Jämsä (19 894)	136	310	15.11	8.61	+ 6.5	2.37	1.30	1.75	< 0.01

The analysis of the data by cause of the mission showed statistically significant increases in violence-related missions and the traffic accident and other accident/injury categories (Table 3). The RR was highest in the violence category (RR 1.87, 95% CI 1.43–2.44) and lowest in the other disease category (RR 1.13, 95% CI 0.90–1.41). The 95% CI was wider in cardiac arrests, as category n was relatively small (Table 3).

**Table 3:** Mission cause analysis

Cause of mission	Event n	Reference n	n/day event	n/day reference	n ± change/day during event	95% CI upper	95% CI lower	RR	Sig.
Other accident / injury	1760	5018	18.14	12.93	+ 5.21	1.76	1.12	1.40	< 0.01
Traffic accident	288	702	2.97	1.81	+ 1.16	2.13	1.26	1.64	< 0.01
Violence	252	540	2.60	1.39	+ 1.21	2.44	1.43	1.87	< 0.01
Cardiac arrest	56	157	0.58	0.40	+ 0.17	2.08	0.98	1.43	0.065
Other diseases	3593	12,758	37.04	32.88	+ 4.16	1.41	0.90	1.13	0.305

The analysis of the mission urgency categories showed that category C missions increased statistically significantly during the events, with an average of 7.13 additional missions per event day (Table 3). The RR analysis of the mission urgency categories showed a RR of 1.25 (95% CI 0.97–1.61) for category A, 1.22 (95% CI 0.97–1.54) for category B and 1.33 (95% CI 1.05–1.66) for category C. The RR for category D was 0.83 (95% CI 0.66–1.04) (Table 4).

**Table 4:** Mission urgency analysis

Mission urgency category	Event n	Reference n	n/day event	n/day reference	n ± change/day during event	95% CI upper	95% CI lower	RR	Sig.
A	345	1102	3.56	2.84	+ 0.72	1.61	0.97	1.25	0.081
B	1385	4525	14.28	11.66	+ 2.62	1.54	0.97	1.22	0.085
C	2816	8498	29.03	21.90	+ 7.13	1.66	1.06	1.33	< 0.05
D	1042	5042	10.74	12.99	− 2.25	1.04	0.66	0.83	0.108

We also investigated mission dispersion by time of day. There was no statistically significant increase in workload during events between 08:00 and 20:00, whereas the RR for 20:00–08:00 h was 1.39 (95% CI 1.11–1.75). Our findings thus indicate that the workload increase during events for EMS and rescue services occurred especially in the evening and at night. The geospatial analysis supported this finding.

## Geospatial analysis

**Fig. 1:**
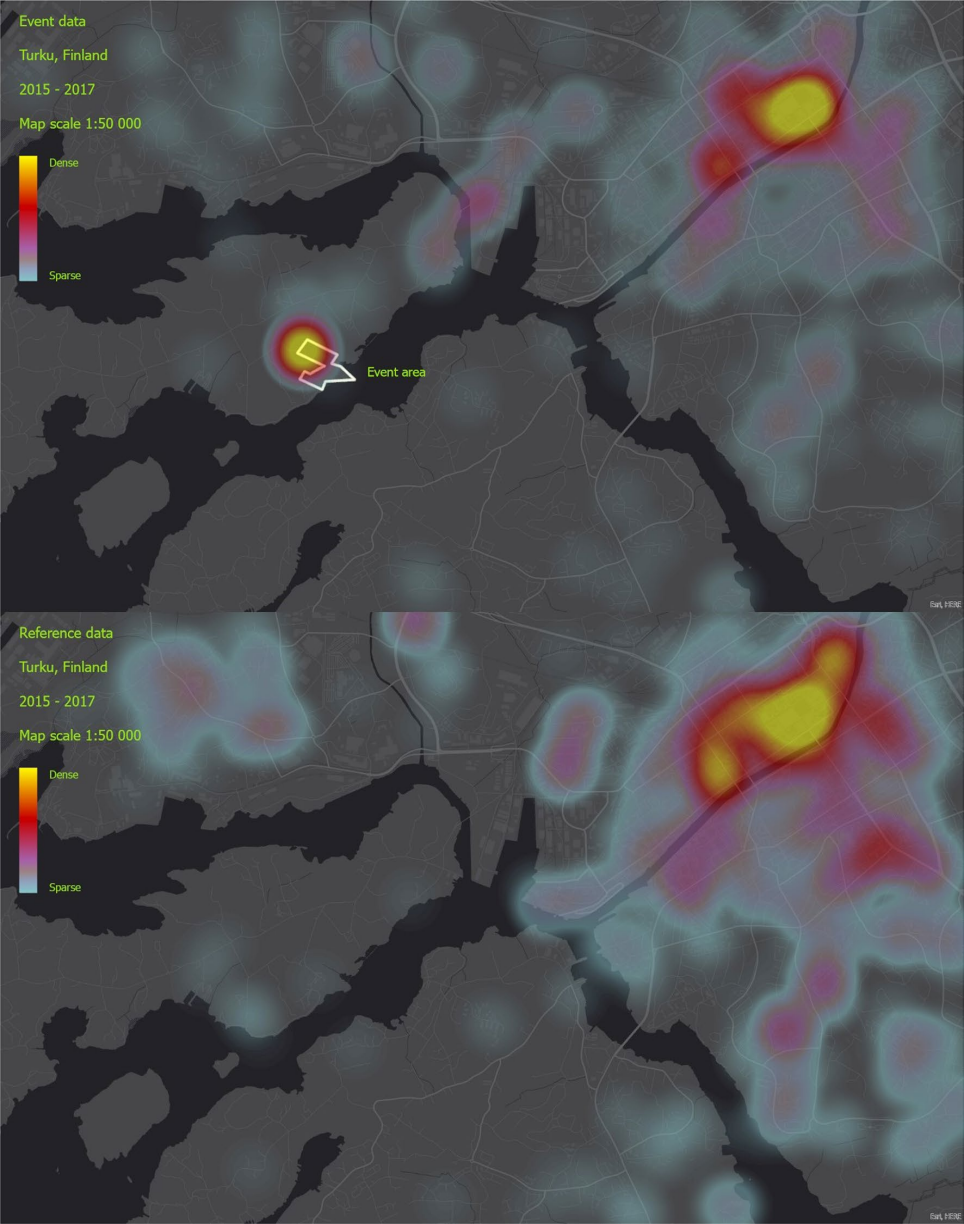
Event/reference data heat map, City of Turku, Finland

The geospatial investigation of the workload during events indicated that the events created dense hotspots of missions in the vicinity of the event areas in addition to common hotspots such as city centres that appeared also in the reference data. This density hotspot is clearly visible in an example illustration from event in Turku (Fig. 1).

In the reference data, the mission density hotspot is only visible in the city centre area, and the event area location is clear. A supplementary mission density animation of an event in Turku shows that missions peaked in the vicinity of the event area, especially during late hours. In addition, missions seemed to occur in the route between the event area and the city centre, near and after the closing time of the event area (Additional file 1: Animation).

## Discussion

Our findings show that the workloads of both the EMS and the rescue services increased during MG events, peaking especially in the evening and at night. The results showed a statistically significant increase in violence-related missions and the traffic accident and other accident/injury categories during the MG events. According to the urgency categories, urgent C-level missions increased the RR and non-urgent category D missions decreased the RR during the events. This affects the initial response efficiency, as urgent missions have shorter response time windows than non-urgent missions. The geospatial analysis revealed hotspots of EMS missions in the vicinity of the event area, as well as an increase in missions between the venue and the city centre. This study revealed information about EMS and rescue services’ workloads during MGs in a completely new level of detail.

Our results confirm previous findings that showed that most missions during MG events are allocated to EMS, whereas the increase in the rescue service workload is minor [1, 6]. Furthermore, previous studies support the finding that MG events impact local healthcare resources [2, 3, 31, 38]. According to our findings, the increase in missions is higher in rural areas than in big cities. This is another aspect that requires attention when considering the need for preparedness, as local resources are typically scaled for their usual population. In this study, the highest increase in workload occurred in the location in which the population was the smallest. As resources in smaller settlements may be more easily overwhelmed than in bigger cities, event organizers’ own levels of preparedness need special focus and attention. This conclusion is supported by previous studies indicating that austere environments also create a need for stronger preparedness for event organizers [6, 30].

A closer investigation of mission causes showed that the RR was highest for violence-related missions. Traffic accidents and other accidents and injuries, including intoxications, also increased. Previous studies have shown that the presence of alcohol and drugs increases the need for medical care [3, 13, 15, 20, 21, 24, 32, 33], whereas on-site medical care [9–13, 31] and alcohol sobering-up facilities [18] decrease the need for outside medical care. A recent study suggested, with an 86% consensus, that in the largest and highest-risk events, event organizers should be required to arrange on-site professional medical services and limit volunteer-based care. However, the expert board did not reach consensus about the event’s professional healthcare operator also participating in the treatment of emergency patients. In other words, treatment of major/life-threatening injuries should be performed by the EMS despite the level of on-site medical care [39]. An increased workload for police is deducible from the high increase in violence-related missions; this has also been recognized in previous studies [1, 4].

The geospatial analysis revealed dense concentrations of EMS missions in the outskirts of the event areas. These hotspots were more visible in the venues outside city centres, as there was also a concentration of missions in the reference data. In addition, in some cases, there was an increase in missions in the geographical area between the event area and the city centre hotspots. Our findings also showed that the workloads peaked at certain times of day, especially at night-time in the vicinity of the event area. According to previous studies, authorities need to pre-allocate additional resources for MG events, but this preparedness should concentrate on the outskirts of the event area [6, 39].

### Strengths and limitations

Using the mission data, we were able to obtain comprehensive information on the EMS and rescue service workloads during MG events. The mission volume, profiles and timestamps were accurate numeric information, which gave more precise results than prior qualitative studies.

Our study included only two of the three major emergency services authorities because police mission data with no EMS participation was not included in the study due to data availability and confidentiality. Despite this limitation, our results indicated an increase in violence-related missions. Investigation of police forces’ workloads could thus give additional valuable information for better overall emergency services preparedness. Unit-specific information was unavailable in our data; therefore, the selection of the responsible authority between the EMS and rescue services was done based on the mission code. In Finland, both EMS and rescue services occasionally respond to each other’s missions. Such mission types are for different types of accidents. In some mission types, such as house fires with no casualties, EMS are dispatched in a work safety role. Rescue services may be used in medical emergencies in the first responder role. Many EMS and rescue service mission types involve both authorities. Therefore, in the general analysis, all missions including both authorities were included in the five-category classification.

The results of this study provide detailed information on the EMS and rescue service workload during MG events. This information can be utilized in the preparedness and resource planning stages of MG events. The use of statistical and geospatial methods enabled the analysis of accurate and adequate resource deployment in the correct geographical locations in the right time window. This effective and sufficient resource deployment enforces the level of service during MG events and may improve opportunities to respond to emergencies.

Outcome measures, such as delay in routine missions and mortality and morbidity during the event, were not included in this study, which may be considered a limitation for the importance of this study. In addition, X-percentage (no transportation to hospital with ambulance) would give valuable and more detailed information on the mission profiles. However, the dataset used in this study contained only city, dispatch time, mission code and GIS information.

## Conclusions

According to this study, EMS and rescue service workloads increase during MG events. The increase focuses mostly on EMS and peaks especially in the evening and at night. The geospatial analysis showed unexpected additional hotspots outside MG event areas during the events. This study is relevant for future similar validation studies internationally.

## Supplementary Information

**Additional file 1.** 24-h workload animation.

## Data Availability

Data are available upon reasonable request from JP.

## Abbreviations

MG: Mass gathering
EMS: Emergency medical services
RR: Relative risk
CI: Confidence interval
